# MicroRNA-27b, microRNA-101 and microRNA-128 inhibit angiogenesis by down-regulating vascular endothelial growth factor C expression in gastric cancers

**DOI:** 10.18632/oncotarget.6059

**Published:** 2015-10-09

**Authors:** Hai-Ting Liu, Ai-Yan Xing, Xu Chen, Ran-Ran Ma, Ya-Wen Wang, Duan-Bo Shi, Hui Zhang, Peng Li, Hong-Fang Chen, Yu-Hong Li, Peng Gao

**Affiliations:** ^1^ Department of Pathology, Qilu Hospital, Shandong University, Jinan, P.R. China; ^2^ Department of Pathology, Qingzhou Center Hospital, Weifang, P.R. China; ^3^ Department of Pathology, Liaocheng Peoples Hospital, Liaocheng, P.R. China

**Keywords:** microRNA-27b, microRNA-101, microRNA-128, angiogenesis, gastric cancer

## Abstract

Vascular Endothelial Growth Factor C (VEGF-C) has critical roles in angiogenesis in human cancers; however, the underlying mechanisms regulating VEGF-C expression remain largely unknown. In the present study, VEGF-C protein expression and the density of blood vessels or lymphatic vessels were determined by immunohistochemistry in 103 cases of gastric cancer tissues. Suppression of VEGF-C by miR-27b, miR-101 and miR-128 was investigated by luciferase assays, Western blot and ELISA. The miRNAs expression levels were detected in human gastric cancers by real-time quantitative PCR. Cell proliferation, migration and invasion assays were performed to assess the effect of miRNAs on gastric cancer cells and human umbilical vascular endothelial cells (HUVECs). Our data showed that high VEGF-C expression was significantly associated with increased tumor size, advanced TNM classification and clinical stage, higher microvessel density (MVD) and lymphatic density (LVD), as well as poor survival in patients with gastric cancer. Furthermore, VEGF-C was found to be a direct target gene of miR-27b, miR-101, and miR-128. The expression levels of the three miRNAs were inversely correlated with MVD. Overexpression of miR-27b, miR-101, or miR-128 suppressed migration, proliferation activity, and tube formation in HUVECs by repressing VEGF-C secretion in gastric cancer cells. We conclude that miR-27b, miR-101 and miR-128 inhibit angiogenesis by down-regulating VEGF-C expression in gastric cancers.

## INTRODUCTION

Gastric cancer is the fourth most common cancer and is one of the leading causes of cancer-related death worldwide [1]. Despite considerable improvements in curative surgery, patients with metastatic gastric cancer still exhibit poor prognoses [2]. For most solid tumors, a multi-step progression to metastasis is the main cause of cancer-related death [3]. Angiogenesis facilitates the initial development of primary malignant neoplasms and progression of metastatic tumors [4]. Likewise, vascular proliferation and angiogenesis are closely involved in infiltration and metastasis of cancer cells [5].

Expression of vascular endothelial growth factor-C (VEGF-C), a member of the platelet-derived growth factor family, correlates significantly with proliferation and migration of vascular endothelial cells, as well as lymphangiogenesis [6]. Furthermore, Kaushal *et al.* [7] and Witte *et al.* [8] found that VEGF-C secreted from cancer cells may directly promote cancer cell migration and invasion by autocrine signaling. Clearly, VEGF-C has important roles in stimulating the development, and possibly the progression of tumors. However, the mechanism regulating VEGF-C expression remains largely unknown.

MicroRNAs (miRNAs) are small, endogenous noncoding regulatory RNAs about 22 nucleotides in length. They usually induce mRNA degradation or suppress translation of the target protein by binding to the 3′untranslated region (3′-UTR) of mRNAs [9]. Recent studies revealed that miRNAs can function as tumor suppressors and be closely correlated with the invasion and metastasis of human cancers, including breast cancer [10], gastric cancer [11], and cutaneous squamous cell carcinoma [12]. Here we investigate whether microRNAs are required in VEGF-C-mediated angiogenesis or lymphangiogenesis in gastric cancers.

## RESULTS

### VEGF-C expression is up-regulated in human gastric cancer tissues

**Figure 1:**
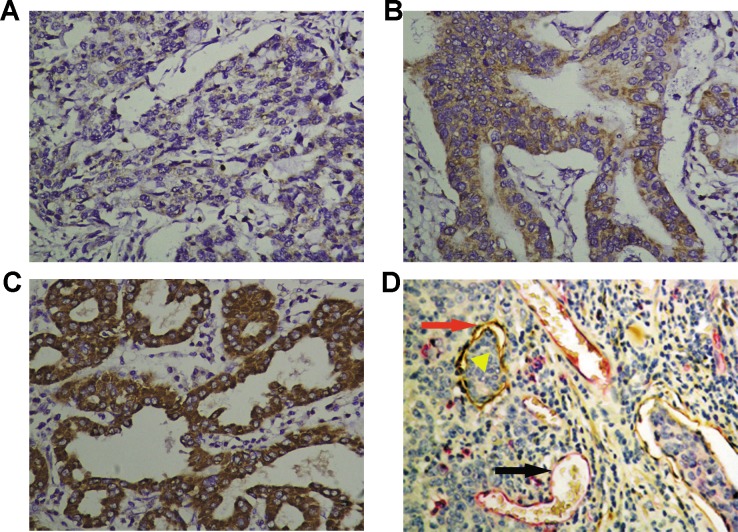
Immunohistochemical staining of VEGF-C, blood vessels, and lymphatic vessels in gastric cancers VEGF-C immunoreactivity was classified as three grades: weak **A.**, moderate **B.** and strong **C.** (× 400). The blood vessels were clearly distinguished from lymphatic vessels by double immunohistochemical staining for CD-34 (red, blank arrowhead) and D2-40 (brown, red arrow). The tumor emboli in the lymphatic vessel was also observed (yellow triangular arrowhead) **D.**, (× 400).

**Figure 2:**
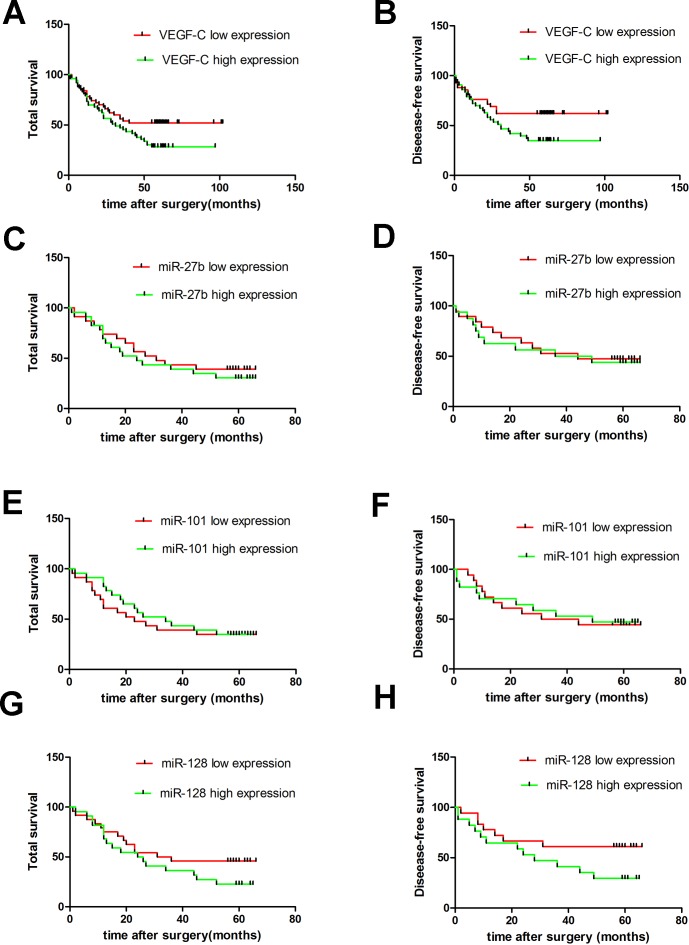
Expression of VEGF-C and miR-27b, miR-101 or miR-128 and their correlation with patients’ survival in gastric cancers Kaplan-Meier survival analysis and Log-rank test showed that the patients with higher VEGF-C expression had a poorer overall survival and disease-free survival. **A.** and **B.** However, there was no significant difference in the overall survival and disease-free survival between the miR-27b **C.** and **D.**, miR-101 **E.** and **F.** or miR-128 **G.** and **H.** lower and higher expression groups.

VEGF-C protein was significantly overexpressed in the cytoplasm of gastric cancers compared to non-tumorous gastric tissues (Figure 1A-1C and Supplementary Figure S8). VEGF-C was highly expressed in 53 cases (51.45%) of gastric cancer, and the other 50 cases (48.55%) showed decreased expression. VEGF-C expression was significantly higher in cases with bigger tumors (*p* = 0.0026), advanced TNM classification (*p* = 0.0383, *p* = 0.0073, or *p* = 0.0232, respectively), or advanced clinical stage (*p* = 0.0078) (Table 1). Moreover, higher expression of VEGF-C was associated with poorer overall survival (*p* = 0.0443) and poorer recurrence-free survival (*p* = 0.0315) (Figure 2A-2B).

**Table 1:** Association of VEGF-C expression with clinicopathological parameters

Variable	n	VEGF-C expression		*p* value
		lower	high	
Age (years)				*p* = 0.1005
<61	51	28	23	
≥61	52	22	30	
Gender				*p* = 0.0660
male	87	45	42	
female	16	5	11	
Tumor size(cm)				*p* = 0.0026
<5	43	28	15	
≥ 5	57	21	36	
Missing	3	1	2	
Clinical stage				*p* = 0.0078
I+II	37	23	14	
III+IV	62	23	39	
Missing	4	4	0	
T classification				*p* = 0.0383
T1	7	6	1	
T2	45	23	22	
T3	38	12	26	
T4	9	5	4	
Missing	4	4	0	
Lymph node metastasis				*p* = 0.0073
N0	24	18	6	
N1	53	19	34	
N2	20	9	11	
N3	2	0	2	
Missing	4	4	0	
Distant metastasis (M)				*p*=0.0232
Negative (M0)	64	35	29	
Positive (M1)	28	9	19	
Missing	11	6	5	
Differentiation				*p* = 0.6184
well	4	1	3	
moderate	30	15	15	
poor	68	34	34	
Missing			1	

### MiR-27b, miR-101, or miR-128 directly down-regulates VEGF-C expression

**Figure 3:**
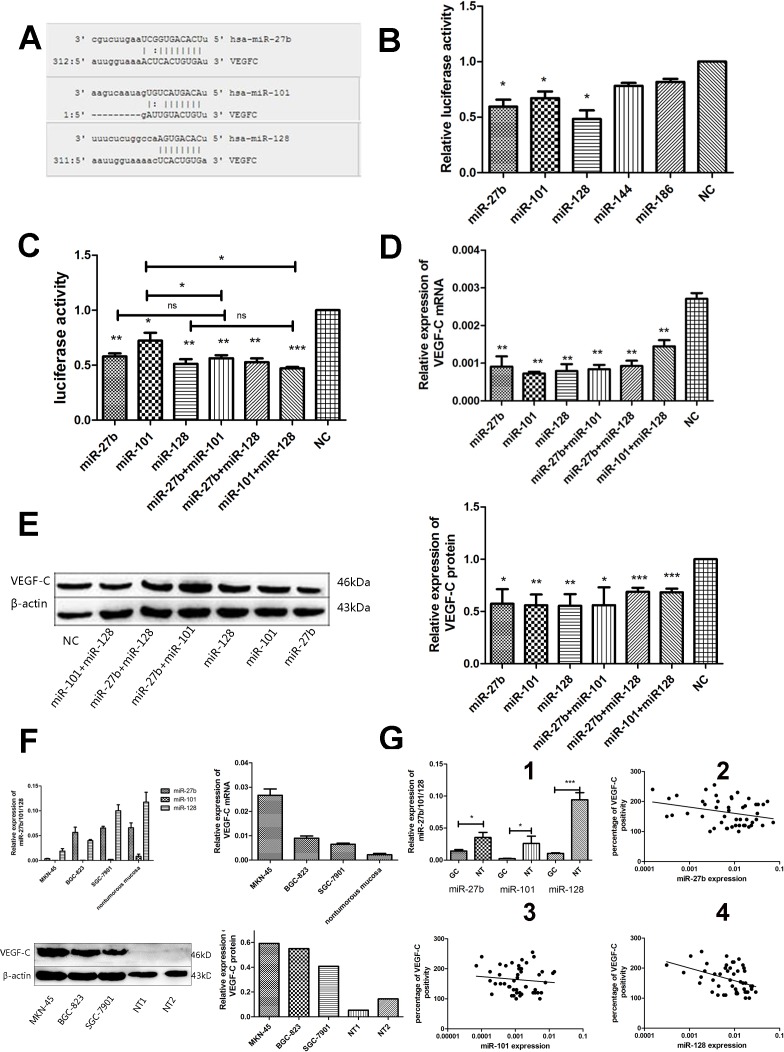
MiR-27b, miR-101, or miR-128 directly down-regulates VEGF-C expression through posttranscriptional repression in gastric cancer cells Scheme representation of the potential binding site of miR-27b, miR-101, or miR-128 in the VEGF-C 3′UTR **A.**. Dual-luciferase reporter gene assay showed that miR-27b, miR-101, or miR-128(decreased 38.68% ± 10.86%, 30.36% ± 10.29%, 47.76% ± 13.61%, *p* = 0.0115, *p* = 0.0156, or *p* = 0.0111) respectively, but not miR-144 or miR-186 displayed strong inhibitory effect on the luciferases expression in MKN-45 cells. **B.**. MiR-27b, miR-101, miR-128, miR-27b/miR-101, miR-27b/miR-128 or miR-101/miR-128 co-transfection could significantly suppress the luciferase activity in pmiR-VEGF-C transfected MKN-45 cells **C.**. MiR-27b, miR-101, miR-128 or miR-27b/miR-101, miR-27b/miR-128, miR-101/miR-128 co-transfection could significantly reduce the VEGF-C mRNA expression in MKN-45 cells **D.**. MiR-27b, miR-101, miR-128 or miR-27b/miR-101, miR-27b/miR-128, miR-101/miR-128 co-transfection could significantly decrease the VEGF-C protein expression in MKN-45 cells **E.**. Compared to human non-tumorous gastric mucosa (*n* = 5), higher expression of VEGF-C mRNA and protein and decreased expression of miR-27b, miR-101 or miR-128 were detected in 3 gastric cancer cell lines by Western blot and RT-qPCR, respectively **F.**. Decreased miR-27b, miR-101, or miR-128 levels were found in gastric cancer tissues compared to the non-tumorous gastric mucosae (G1). An inverse correlation was found between miR-27b (G2) and miR-128 (G4) expression and VEGF-C levels in human gastric cancers samples. However, there was no significantly correlation between miR-101 level and VEGF-C expression (G3). *P < 0.05, **P < 0.01, ***P < 0.001 ns = nonsignificant.

The luciferase assays showed that miR-27b, miR-101, or miR-128 rather than miR-144 or miR-186 (Figure 3A and 3B) displayed more effectively inhibited luciferase activity with an inhibitory rate of more than 30% in pmiR-VEGF-C and miRNAs co-transfected cells, indicating that miR-27b, miR-101, and miR-128 were candidate miRNAs for VEGF-C. Specifically, miR-27b, miR-101, or miR-128 transfection decreased luciferase expression by 41.65 ± 4.60%, 30.36 ± 15.99%, and 51.20 ± 7.3%, respectively in MKN-45 cells (Figure 3C, *p* = 0.0020, *p* = 0.0179, or *p* = 0.0037). The three miRNAs dramatically attenuated the VEGF-C mRNA expression by 69.26% ± 13.87%, 73.24% ± 0.47%, or 72.26% ± 14.97% in MKN-45 cells (Figure 3D, *p* = 0.0052, *p* = 0.0033, or *p* = 0.0021), respectively. The efficacy of miR-27b, miR-101, and miR-128 was significant as indicated by reduced VEGF-C protein levels (36.38 ± 27.62%, 40.56 ± 20.50%, or 43.22 ± 22.27% reduction) (Figure 3E, *p* = 0.0108, *p* = 0.0026, or *p* = 0.0036, respectively). Down-regulation of VEGF-C expression was further verified in the cell line SGC-7901 (Supplementary Figure S1B-D).

We evaluated 48 samples of gastric cancer tumors for expression of miR-27b, miR-101, and miR-128. Levels of all three miRNAs were down-regulated in gastric cancers compared to non-tumorigenic gastric mucosae (Figure 3G1 *P* = 0.0133, *P* = 0.0298, or *P* < 0.0001). Additionally, miR-27b and miR-128 expression inversely correlated to VEGF-C expression in gastric cancers (Figure 3G2 and 3G4 *p* = 0.0492, *r* = −0.2414 or *p* = 0.0031, *r* = −0.3900), although there was no statistically significant inverse correlation between miR-101 level and VEGF-C expression (Figure 3G *P* = 0.3224, *r* = −0.068). Interestingly, expression of the three miRNAs did not correlate with patient prognosis (Figure 2C-2H). However, levels of the three miRNAs decreased in cancer cell lines, which was associated with a corresponding increase in VEGF-C expression (Figure 3F). Importantly, cell lines with higher miR-27b, miR-101, or miR-128 expression tended to have lower VEGF-C expression, while cell lines with lower expression of miR-27b, miR-101 or miR-128 had higher VEGF-C expression. Collectively, our data suggest VEGF-C is down-regulated by miR-27b, miR-101, or miR-128.

Next, we investigated whether the three miRNAs synergistically down-regulated VEGF-C expression. Gastric cancer cells were co-transfected with miR-27b and miR-101, miR-27b and miR-128, or miR-101 and miR-128. Our data showed that miR-27b and miR-101, miR-27b and miR-128, or miR-101 and miR-128 co-transfection led to significant decreases in luciferase activity (miR-27b and miR-101: 42.58% ± 4.83%, *p* = 0.002; miR27b and miR-128: 45.59% ± 5.99%, *p* = 0.0027; miR101 and miR-128: 53.39% ± 2.27%, *p* = 0.0003), decreases of VEGF-C mRNA expression by 70% ± 4.50%, 66.55% ± 5.67%, or 47.18% ± 5.4% (*p* = 0.0012, *p* = 0.0017, or *p* = 0.0058, respectively) and decreases of VEGF-C protein expression by 52.92% ± 33.83%, 34.04% ± 7.59%, or 31.94% ± 6.99% (*p* = 0.0205, *p* < 0.0001, or *p* < 0.0001, respectively) in MKN-45 cells (Figure 3C-3E). Although co-transfection resulted in significant suppression of VEGF-C, suppression was not significantly higher than individual miRNA transfection, suggesting that the three miRNAs do not have synergistic activities.

### MiR-27b, miR-101, or miR-128 suppresses the migration or invasion activity of gastric cancer cells *in vitro*

**Figure 4:**
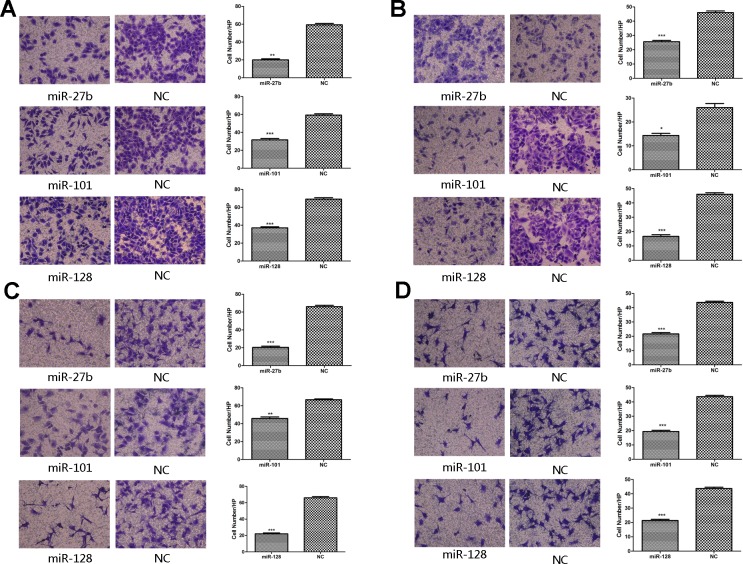
Overexpression of miR-27b, miR-101, or miR-128 abolished the migration, invasion activity of gastric cancer cells and the migration activity of HUVECs *in vitro* In migration assay, the migration activity of the miRNAs-transfected MKN-45 cells was significantly decreased when compared to the negative control **A.**. Matrigel invasion assay showed reduced invasion capabilities of the miRNAs-transfected MKN-45 cells than those of the negative control cells **B.**. The cell culture supernatant from the miRNAs-transfected groups rather than the negative control groups inhibited the migration capabilities of HUVECs in both MKN-45 **C.** and SGC-7901 cells **D.**. *P < 0.05, **P < 0.01, ***P < 0.001.

Previous studies suggest the VEGF-C/VEGFR-3 axis is critical in enhancing cancer cell migration and invasion and promotes metastasis [13]. Based on these studies, we investigated whether miR-27b, miR-101, and miR-128 could inhibit migration and invasion. As shown in Figure 4A, miR-27b, miR-101, or miR-128-transfected cells showed a considerable decrease in migration activity by 64.9% ± 4.27%, 45.16% ± 3.71%, or 46.38% ± 0.56% (*p* = 0.0001, *p* < 0.0001, or *p* = 0.0003, respectively) and a decrease in invasion capacity by 45.83% ± 4.83%, 39.13% ± 9.46% or 63.64% ± 3.49% (Figure 4B *p* = 0.0004, *p* = 0.0133, or *p* = 0.0002, respectively) than that of the negative control group in MKN-45 cells. In addition, transfection of the three miRNAs also decreased the migration or invasion abilities of cell line SGC-7901 (Supplementary Figure S2-S3). Collectively, these results suggest ectopic expression of miR-27b, miR-101 or miR-128 significantly inhibits migration and invasion activity of gastric cancer cells *in vitro*, and provide further evidence that metastasis due to VEGF-C is likely due to downregulation of key miRNAs.

### MiR-27b, miR-101, or miR-128 suppresses the migration and proliferation activity in HUVECs

**Figure 5:**
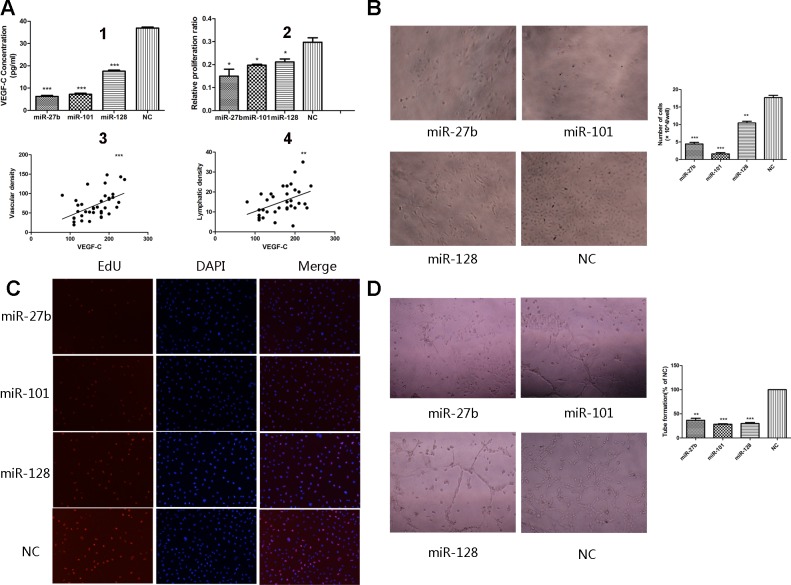
Overexpression of miR-27b, miR-101, or miR-128 attenuated proliferation and tube formation of HUVECs The secretion level of VEGF-C significantly decreased in the culture supernatant from the miRNA-transfected cells, compared to the negative control groups, determined by ELISA (A1). The percentages of proliferating cells were quantified by EdU incorporation experiments. Decreased proliferation activity of the miRNAs-transfected groups was observed compared to the negative control groups. (A2). Significantly inverse correlation was found between VEGF-C expression and MVD (A3) or LVD (A4) in human gastric cancers samples. The number of HUVECs decreased by treatment with the medium supernatant from the miRNAs-transfected groups compared to the negative groups **B.**. Proliferating HUVECs were labeled after conjugated reaction of Apollo dye and EdU (red). Cell nuclei stained with DAPI (blue) represents a total population of cells. The images are representative of the results obtained **C.**, with the quantifiable results shown in Figure 5A2. Tube formation of endothelial cells was dramatically inhibited in the transfected groups compared to the negative groups **D.**. *P < 0.05, **P < 0.01, ***P < 0.001.

The process that VEGF-C stimulates proliferation and migration of vascular endothelial cells is a critical in angiogenesis. To determine whether miR-27b, miR-101, or miR-128 inhibits VEGF-C-induced endothelial cell migration, transwell monolayer permeability assays were used to detect the changes in migration activity of HUVECs, which were treated with the culture supernatants of gastric cancer cells transiently transfected with the three miRNAs or a negative control. Inhibition of miR-27b, miR-101, or miR-128 affected VEGF-C secretion was confirmed by ELISA. VEGF-C levels were significantly reduced by 82.23% ± 2.07%, 81.54% ± 1.76%, or 52.33% ± 1.94% respectively in MKN-45 cells transfected with miR-27b, miR-101, or miR-128 (Figure 5A1 and Supplementary Figure S4B) (all *p* < 0.05). Transwell chamber migration assays on HUVECs showed a significantly attenuated migration capability in cells transfected with miRNAs compared to cells transfected with a negative control (Figure 4C and 4D, all *p* < 0.01). Additionally, HUVECs had reduced proliferation when incubated with culture supernatants from cells transfected with the three miRNAs (Figure 5B and Supplementary Figure S4A, all *p* < 0.01). In addition, EdU analysis showed that the proliferation rate of HUVECs decreased by 57.14% ± 20.72%, 32.03% ± 8.15%, or 29.46% ± 2.24%, respectively, in the transfected groups compared to that of the negative groups (Figure 5A2 and 5C, *p* = 0.0132, *p* = 0.0182, or *p* = 0.0175). Comparable results were observed with HUVECs incubated with culture supernatants harvested from SGC-7901 cells transfected with miRNAs (Supplementary Figures S4A and S5). Collectively, these data suggest miR-27, miR-101, and miR-128 suppress the migration and proliferation of HUVECs at least in part by down-regulating VEGF-C secretion.

### MiR-27b, miR-101, or miR-128 suppresses the tube formation of HUVECs

We used a three-dimensional Matrigel assay to determine the importance of miR-27b, miR-101, or miR-128 on VEGF-C-induced tube formation, which was another significant step in the angiogenic process. As expected, the tube formation (elongated tube-like structures) of endothelial cells was significantly inhibited when treated with supernatants from the miRNAs-transfected MKN-45 cells compared to cells transfected with a negative control group (Figure 5D, *p* = 0.0001, *p* = 0.0005, or *p* = 0.0001, respectively). Comparable results were observed with supernatants harvested from SGC-7901 cells transfected with miRNAs (Supplementary Figure S6). These results suggest that miR-27b, miR-101, or miR-128 attenuates tube formation of HUVECs induced by secreted VEGF-C from gastric cancer cells *in vitro*.

To determine whether expression of the three miRNAs or VEGF-C correlates with MVD or LVD in human gastric cancers, double immunohistochemical staining for CD34/D2-40 was done to quantitatively evaluate the number of blood vessels (CD34 positive) or lymph vessels (D2-40 positive) in gastric cancer samples (Figure 1D). Importantly, VEGF-C expression positively correlated with MVD and LVD (Figure 5A3 and A4 *p* = 0.0003 or *p* = 0.0027), and miRNA-27b, miR-101, or miR-128 levels inversely correlated with MVD (Supplementary Figure S7A-C *p* = 0.0471, *p* = 0.0442, or *p* = 0.0018); no correlation between the level of the three miRNAs and LVD was found (Supplementary Figure S7D-F *p* > 0.05).

## DISCUSSION

VEGF-C, a member of the VEGF family, can stimulate the proliferation and migration of both vascular and lymphatic endothelial cells [6, 14]. Additionally, VEGF-C autocrine signaling is critical for tumor cell migration and invasion [15]. Our study supports previous works and demonstrates that gastric cancer tissues and cell lines have elevated VEGF-C. We also show that VEGF-C expression is correlated with MVD and LVD, which are necessary for angiogenesis and lymphangiogenesis in gastric cancer.

So far, several recent studies demonstrated that DNA methylation, histone modification, transcription factor and other genes were involved in the regulation of VEGF-C. Da *et al*. demonstrated that S-adenosylmethionine (SAM), an inhibitor of intracellular demethylase activity, enhanced VEGF-C methylation levels and thus suppressed its gene expression [16]. Additionally, suberoylanilide hydroxamic acid (SAHA), a potent histone deacetylase (HDAC) inhibitor, suppresses VEGF-C transcription [17]. Moreover, Engelmann *et al.* reported that E2F1, which were required for S-phase progression, up-regulated VEGF-C/VEGFR-3 expression to promote angiogenesis by binding and activating VEGFR-3 and VEGF-C gene promoters [18]. Additionally, Sun *et al.* reported that metastasis-associated in colon cancer-1 (MACC1) increased expression of VEGF-C by affecting HGF/c-Met signaling pathway, and thus promoted lymphangiogenesis in human gastric cancer [19]. Besides that, microRNAs could have crucial roles in tumorigenesis and progression of human tumors [20]. MiRNAs may function in suppressing expression of oncogenes or tumor suppressors. We hypothesized that overexpression of VEGF-C in gastric cancers is due to reduced expression of one or several miRNAs. We identified three miRNAs that target and suppress VEGF-C: miR-27b, miR-101, or miR-128. Importantly, we found that miR-27b and miR-128 are significantly down-regulated and inversely correlated with VEGF-C expression in gastric cancer cell lines and tissues. MiR-101 was also down-regulated in gastric cancer tissues and cell lines; however, there was no significant inverse correlation between its expression level and VEGF-C expression in gastric cancer tissues. Additionally, compared to miR-27b and miR-128, miR-101 was less efficient at reducing luciferase activity indicating it is less specific for VEGF-C mRNA. Thus, we believe that miR-101 inhibits VEGF-C expression by a fine-tuning manner.

Our results support previous studies implicating the three miRNAs in tumorigenesis and progression. Ye *et al*. demonstrated that miR-27b suppressed tumor growth and angiogenesis in colorectal cancer [21]. MiR-101 suppressed bladder cancer cell migration and invasion by down-regulating VEGF-C expression [22]. Similarly, the expression of VEGF-C was down-regulated by miR-128 in non-small cell lung cancer [23]. However, few studies have addressed the role of these three miRNAs in human gastric cancers and none have correlated miRNA expression with MVD, VEGF-C expression, or clinical data. Here we demonstrated that miR-27b, miR-101, and miR-128 inhibited HUVEC migration, proliferation and tube formation by reducing secretion of VEGF-C by gastric cancer cells. Additionally, we showed that levels of the three miRNAs were inversely correlated with MVD in human gastric cancer specimens. Collectively, these findings highlight the role of miRNAs in suppressing carcinogenesis, tumor development, and progression and suggest that aberrant overexpression of VEGF-C may be due to the decreased miR-27b, miR-101, or miR-128 expression in gastric cancers.

A recent study showed that miR-143 and miR-145 synergistically inhibit ERBB3 expression in breast cancer [24]. Additionally, miR-424 and miR-381 synergistically target WEEN-1 in renal carcinoma [25]. However, in the present study, we did not observe a synergistic effect of any two miRNAs in suppressing VEGF-C expression. As the results showed, miR-101 merely restrained its expression by about 30% via a fine-tuning manner; however, miR-27b or miR-128 has inhibited VEGF-C expression by ∼45% or more respectively. So, our data didn't show a significant difference in the suppressive effect on VEGF-C expression between the two miRNAs co-transfection group and individual miRNA group. Similarly, Derfoul *et al*. demonstrated that miR-101 and miR-214 co-operatation could not reduce Ezh2 levels further while both miR-214 and miR-101 could suppress Ezh2 protein level [26]. They also suggested that Ezh2 overexpression may also arise from deletion of miR-214 allele without concomitant deletion of miR-101 in a subset of breast tumors. Other mechanisms underlying the synergistic effect of miR-27b, miR-101 and miR-128 on VEGF-C expression need further investigation in the future. In addition to reduced VEGF-C expression in gastric cells transfected with miR-27b, miR-101, or miR-128, migration and invasion abilities were also attenuated, indicating that autocrine regulation of gastric cancer cells is critical for tumorigenesis [8].

The miRNA-transfected cells secreted less VEGF-C. Consistent with these results, HUVECs cultured with supernatant from the miRNA-transfected cells had reduced ability to form tubes and reduced migration and proliferation. We also found that levels of the three miRNAs were inversely correlated with MVD. Additionally, VEGF-C expression positively correlated with MVD in human gastric cancer. These data suggest the three miRNAs are important in preventing angiogenesis in gastric cancers. VEGF-C expression was also positively correlated with LVD in the study. However, there was no significant association between the levels of the three miRNAs and LVD. Effects of VEGF-C on lymphatic endothelial cells’ (LECs’) proliferation, migration, and whether the three miRNAs suppress lymphangiogenesis in gastric cancers need to be investigated later.

This study extends our knowledge about the regulation of VEGF-C expression and angiogenesis by miRNAs, and may help in understanding the molecular mechanisms of gastric cancer's development and progression. Despite that, many challenges on the long way of transiting miRNAs as potential diagnostic or prognostic biomarker still remain from bench to bedside. First, convincing evidences showed that one miRNAs generally had multiple target genes, and more than 50% of mRNAs were frequently down-regulated by multiple miRNAs [27]. The complexities of the network make miRNAs unspecific in regulating multistep process leading to gastric cancers. Moreover, in the experiment, we used miRNA mimics that simulated biological endogenous miRNAs. However, the effect of miRNA mimics could not be maintained stably *in vivo*. The effects of lentivirus packing vectors that can overexpress miRNAs stably *in vivo* need to be investigated further in the future. In spite of many difficulties, the potential applicability of the three miRNAs as a new class of cancer biomarkers is promising and cannot be underrated.

## MATERIALS AND METHODS

### Patient tissue samples

Paraffin-embedded specimens from 103 cases of gastric cancers and 50 samples of non-tumorous gastric mucosae adjacent to carcinomas were obtained from the Qi Lu Hospital of Shandong University. Detailed clinical and pathologic data were collected and are provided in Table 1, including tumor size, TNM classification (UICC, 2002), clinical stage, etc. All samples were confirmed by 2 well-trained pathologists. The collection of tissue samples was obtained with patients’ informed consent. Approval to conduct this study was obtained from the Ethics Committee of Shandong University, China.

### Immunohistochemistry (IHC) staining for VEGF-C

IHC was performed as previously described [28]. Paraffin-embedded gastric cancer sections were incubated with antibody specific for VEGF-C (Zhongshan Goldenbridge Biotechnology, Beijing, China). Cytoplasmic immunoreactivity in tumor cells was considered positive. VEGF-C expression was semi-quantitatively evaluated as previously reported [29]. VEGF-C staining intensity was classified as three grades: weak (1 point), moderate (2 points), or strong (3 points). A staining score was calculated as follows: score (maximum of 300) = sum of 1 × percentage of weak, 2 × percentage of moderate and 3 × percentage of strong staining. VEGF-C immunoreactivity was classified as high or low expression based on a median score.

### Double IHC staining for CD-34/D2-40

Double IHC staining for CD34 (Sangon, China) and D2-40(Zhongshan Goldenbridge Biotechnology, Beijing, China) was done to evaluate the number of the blood vessels (CD34) and lymph vessels (D2-40) as previously reported [30]. The microvessel density (MVD) and lymph vessel density (LVD) were calculated as described previously [28]. The mean MVD and LVD were calculated for each case.

### Cell culture and transfection

The human gastric cancer cell lines MKN-45, SGC-7901, BGC-823 were purchased from the Shanghai Cancer Institute. Human umbilical vein endothelial cells (HUVECs) were generously provided by Dr. Hu (Shandong University). All cells were routinely grown in RPMI 1640 supplemented with 10% heat-inactivated fetal bovine serum (FBS, Gibco BRL, Grand Island, NY, USA). Gastric cancer cells were transiently transfected with miRNA mimics or the negative control (GenePharma, Shanghai, China) by using X-tremeGENE transfection reagent (Roche Applied Science, Indianapolis, IN, USA). Cells were collected 48 h after transfection for RNA and protein extraction. Transfected cells showed overexpression of miRNAs compared to cells transfected with the negative control (Supplementary Figure S1E and F).

### Real-time quantitative RT-PCR

MicroRNA extraction in human gastric cancer samples was performed using a miRNeasy FFPE kit (Bioteke, China). RT-PCR was performed using an All-in-One™ miRNA qRT-PCR Detection Kit (Genecopoeia, USA) with specific miR-27b, miR-101, or miR-128 primers (Genecopoeia, USA). For cell lines, total RNAs were extracted using Trizol Agent (Invitrogen, Carlsbad, CA, USA). The relative expression level of VEGF-C or the three miRNAs was normalized to a control gene and analyzed by the 2^−ΔCt^ method [31].

### Western blot

Forty-eight hours after transfection with the miRNA mimics or the negative control, total protein was extracted from the transfected cells, incubated with primary antibodies against VEGF-C (1:300, Sangon) or β-actin (1:500, Zhongshan) overnight at 4°C, washed, and then detected with a chemiluminescence kit (Millipore, Billerica, MA, USA) according to the manufacturer's procedure.

### Prediction of miRNAs targeting VEGF-C

To explore the regulatory mechanism of VEGF-C in gastric cancer, prediction algorithms, including TargetScan (http://genes.mit.edu/tscan/targetscan2003.html), miRWalk (http://www.umm.uni-heidelberg.de/apps/zmf/mirwalk/) and microRNA.org (http://www.microrna.org/microrna/home.do) were used to screen miRNAs that potentially bind to the 3′-UTR of VEGF-C. Potential miRNAs were picked based on their reported functions in the literature and the number of predicted target sites. Five tumor-suppressing miRNAs including miR-27b, miR-101, miR-128, miR-144, and miR-186, which have potential binding sites in the 3′-UTR of VEGF-C (Figure 3A and Supplementary Figure S1A), were selected for further investigation.

### Dual-luciferase 3′ UTR-reporter assay

The vector for expression of human VEGF-C's 3′ UTR was purchased from RiboBio Co. Ltd (amplified by PCR and inserted between the restrictive sites Xho I and Not I of firefly/Renilla luciferase reporter vector pmiR-RB-REPORT, named pmiR-VEGF-C). MKN-45 and SGC-7901 cells were cultured in 12-well plates, transiently co-transfected with the reporter plasmids and miRNA mimics or a negative control using Lipofectamine 2000 (Invitrogen, USA). Forty-eight hours after transfection, lysates were collected. Relative luciferase activity was measured using a luciferase assay kit (Promega) according to the dual-luciferase assay manual.

### Migration assay and invasion assay in gastric cancer cells

Migration assays were performed with Transwell inserts containing a polycarbonate membrane with 8.0 μm pores (Corning, New York, NY, and USA). For matrigel invasion assays, membranes were coated with Matrigel matrix (BD Science, USA). Twenty-four hours post-transfection with the miRNA mimics or the negative control, gastric cancer cells (5 × 10^5^) in 200 μl serum-free media were added into the upper chamber. Media containing 600 μl of 10% fetal bovine serum served as the chemoattractant in the lower chamber. Cells were incubated for 24 h at 37°C in a CO_2_ incubator. The non-migrating or non-invading cells on the upper surface of the membrane were removed with a cotton swab, while the migrated or invaded cell attached to the bottom of the membrane insert were fixed, stained, and then counted under the microscope (Olympus, Japan).

### Enzyme-linked immunosorbent assay (ELISA)

Cell culture supernatants of gastric cancer cells transiently transfected with the miRNA mimics or the negative control were collected and centrifuged at 3000 rpm for 5 min. Clarified supernatants were collected and VEGF-C secretion levels were determined by ELISA (Cusabio Biotech Co., Ltd).

### Proliferation and migration assays in HUVECs

MKN-45 and SGC-7901 cells were transiently transfected with miRNA mimics or the negative control. Forty-eight hours after transfection, the cell culture supernatant was collected and the VEGF-C secretion level was detected by ELISA. HUVECs were added to twelve-well plates (10^4^ cells/well) in the cell culture supernatant. For cell proliferation assays, cells were counted and photographed under the microscope on the fifth day (Olympus, Japan). For cell migration assays, HUVECs (3 × 10^4^/well) in 200 μl serum-free medium were added to the upper chamber. Cell culture supernatant (600 μl) from MKN-45 or SGC-7901 cells was added to the lower chamber and HUVECs were treated as previously reported [32].

### EdU cell proliferation assay

Proliferation of HUVECs was detected using the cell-light 5-ethynyl-20-deoxyuridine (EdU) Apollo Imaging Kit (RiboBio, China). HUVECs cultured with the media supernatant from cancer cells transfected with miR-27b, miR-101, miR-128 mimics, or negative control, were incubated with 50 μM EdU for 4 h. Samples were fixed, permeabilized, and stained for EdU. Nucleic acids in all cells were stained with 4′, 6-diamidino-2-phenylindole (DAPI). Proliferating cells were stained by Apollo dye and EdU, resulting in red fluorescence. All images were obtained by a fluorescence microscope (Olympus, Japan).

### Tube formation assay *in vitro* (angiogenesis assay *in vitro*)

The ability of HUVECs to form tubes on Matrigel (BD Sciences, USA) was assessed as previously reported [33], and evaluated using an inverted phase contrast microscope. Tubular structures were quantified by manually counting under low-power fields as previously described [34].

### Statistical analysis

A student's *t*-test was performed to analyze differences between two groups. The chi-square test analysis was applied for the correlation between VEGF-C expression and individual clinical pathological parameters. The overall or disease-free survival rates were assessed by the Kaplan and Meier method and the differences between the survival curves were analyzed by the log-rank test. Spearman's correlation was applied for analyzing the association between miR-27b, miR-101, or miR-128 and VEGF-C expression, MVD or LVD, as well as the association between VEGF-C expression and MVD or LVD. The statistical significance of the differences is given as *p* < 0.05. All statistical analyses were performed with GraphPad PrismTM version 5.00 software (GraphPad Software, USA).

## SUPPLEMENTARY MATERIAL FIGURES


