# Supplementary material for: MicroRNA-27b, microRNA-101 and microRNA-128 inhibit angiogenesis by down-regulating vascular endothelial growth factor C expression in gastric cancers
Source: Oncotarget. 2015 Oct 9;6(35):37458–70. doi: 10.18632/oncotarget.6059 (PMC4741941; doi:10.18632/oncotarget.6059)
Supplement: Supplementary file 1 [file oncotarget-06-37458-s001.pdf]

MicroRNA-27b, microRNA-101 and microRNA-128 inhibit angiogenesis by down-regulating vascular endothelial growth factor C expression in gastric cancers

Supplementary Material

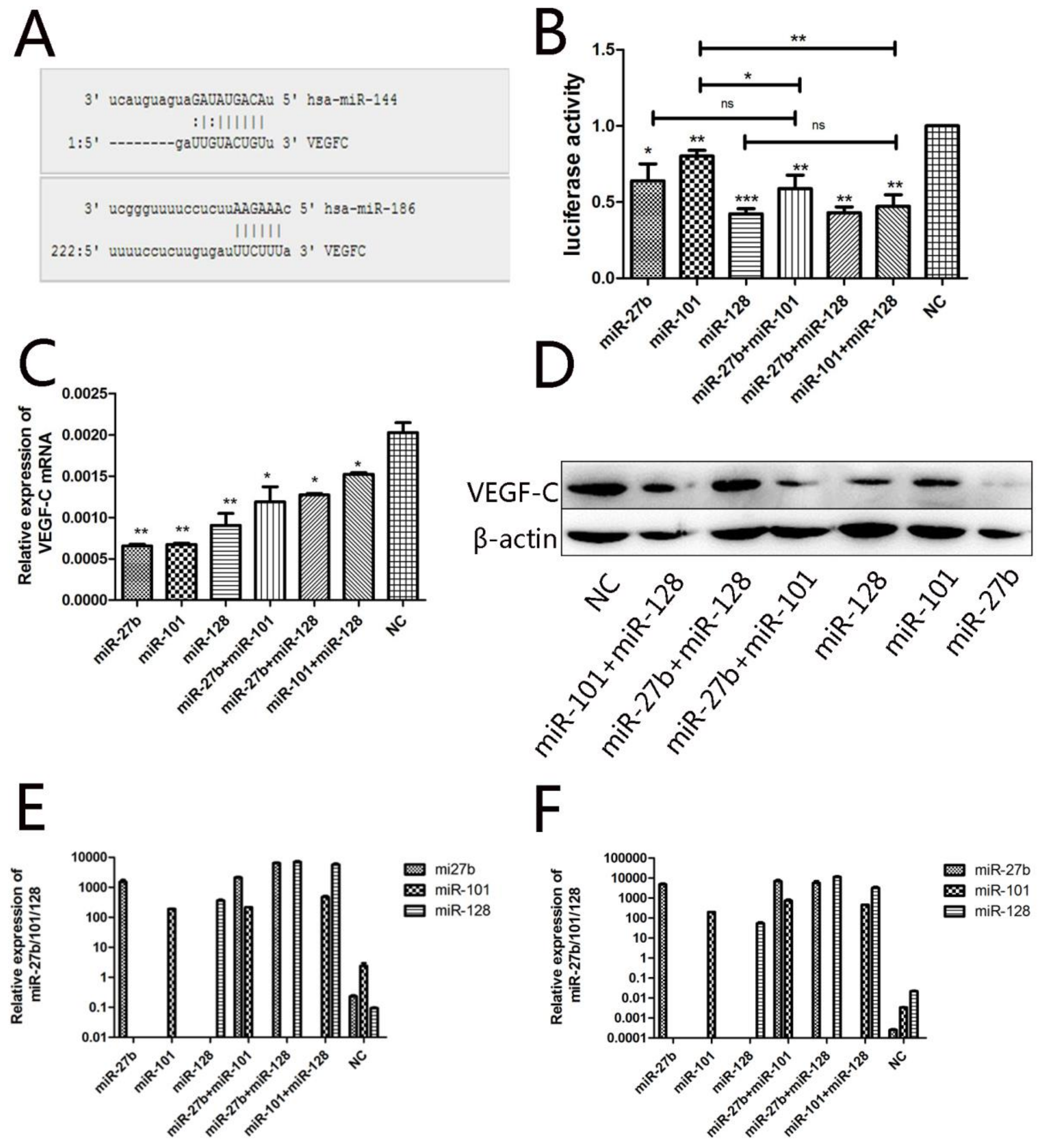

Figure S1. Scheme representation of the potential binding site of miR-144 or miR-186 in the VEGF-C 3 ’-UTR (A). MiR-27b, miR-101, miR-128,

miR-27b/miR-101, miR-27b/miR-128 or miR-101/miR-128 co-transfection could significantly suppress the luciferase activity in pmiR-VEGF-C transfected SGC-7901 cells (B). MiR-27b, miR-101, miR-128 or miR-27b/miR-101, miR-27b/miR-128, miR-101/miR-128 co-transfection could significantly reduce the VEGF-C mRNA expression in SGC-7901 cells (C). MiR-27b, miR-101, miR-128 or miR-27b/miR-101, miR-27b/miR-128, miR-101/miR-128 co-transfection could significantly decrease the VEGF-C protein expression in SGC-7901 cells (D). MiR-27b, miR-101 or miR-128 was successfully overexpressed in MKN-45(E) and SGC-7901(F) cells transfected the three miRNAs group compared to the negative group at 48h after the transfection.

*$P < 0.05$, **$P < 0.01$, ***$P < 0.001$ ns = nonsignificant

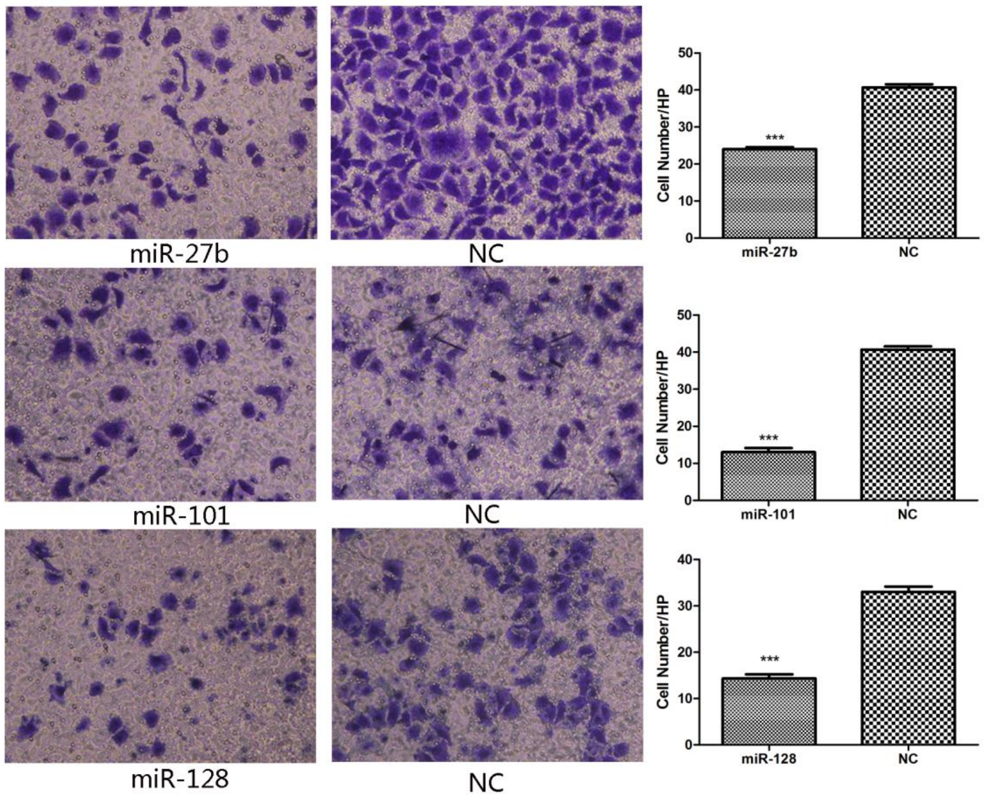

Figure S2. Overexpression of miR-27b, miR-101 or miR-128 abolished the migration activity by 41.46% ± 4.69%, 69.05% ± 5.89%, or 57.58% ± 2.05% in the SGC-7901 cells(p = 0.0003, p = 0.0002, or p = 0.0005) respectively.

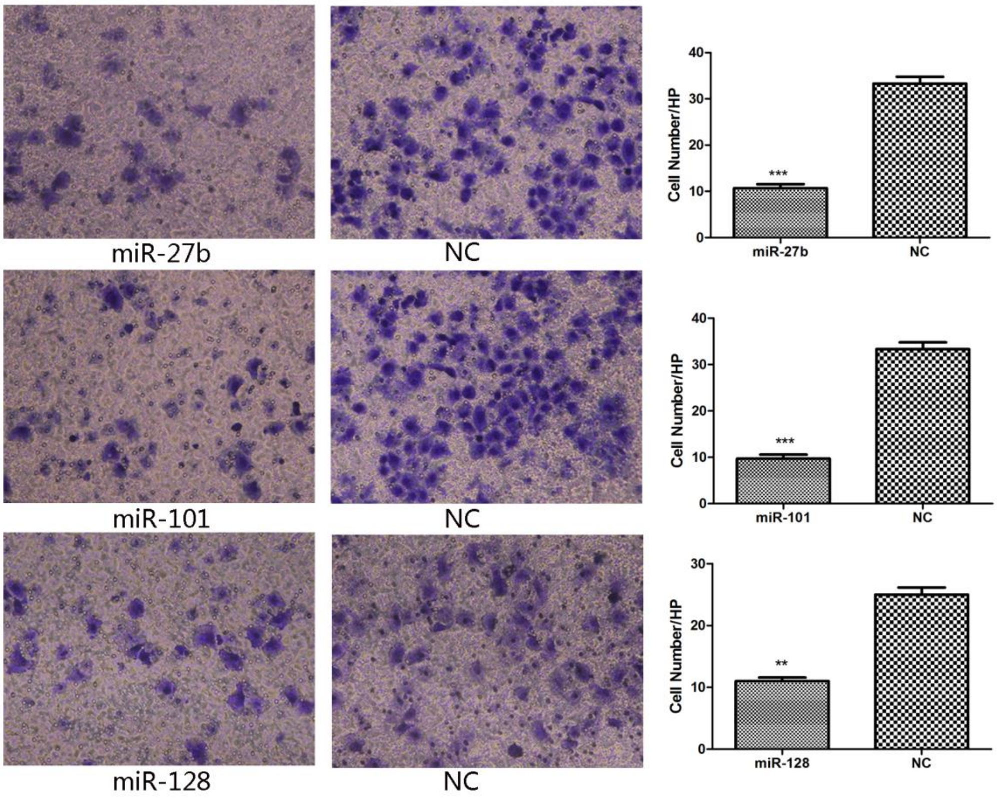

Figure S3. Matrigel invasion assay showed reduced invasion capabilities by 69.44% ± 3.86%, 72.22% ± 3.96% or 52.17% ± 6.28% in the miRNAs-transfected SGC-7901 cells compared to those of the negative control cells (p = 0.0005, p = 0.0004, or p = 0.0042) respectively.

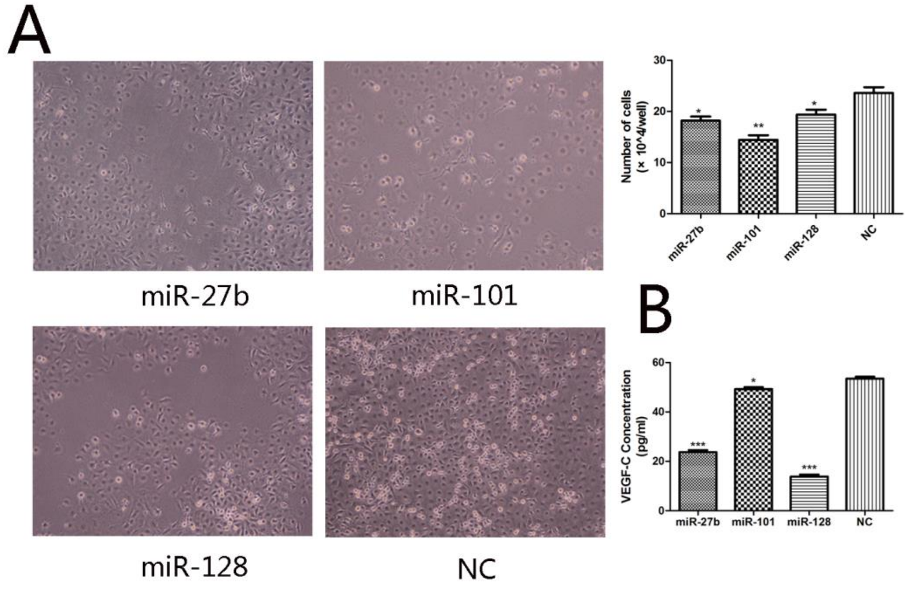

Figure S4. The number of HUVECs decreased by 24.2% ± 3.16%, 38.52% ± 2.51%, or 19.07% ± 2.49% in the miRNAs-transfected SGC-7901 cells compared to those of the negative control cells(p = 0.0144, p = 0.0038, or p = 0.0311) respectively(A). The secretion level of VEGF-C significantly decreased in the culture supernatant from the miRNA-transfected SGC-7901cells, compared to the negative control cells, determined by ELISA (B).

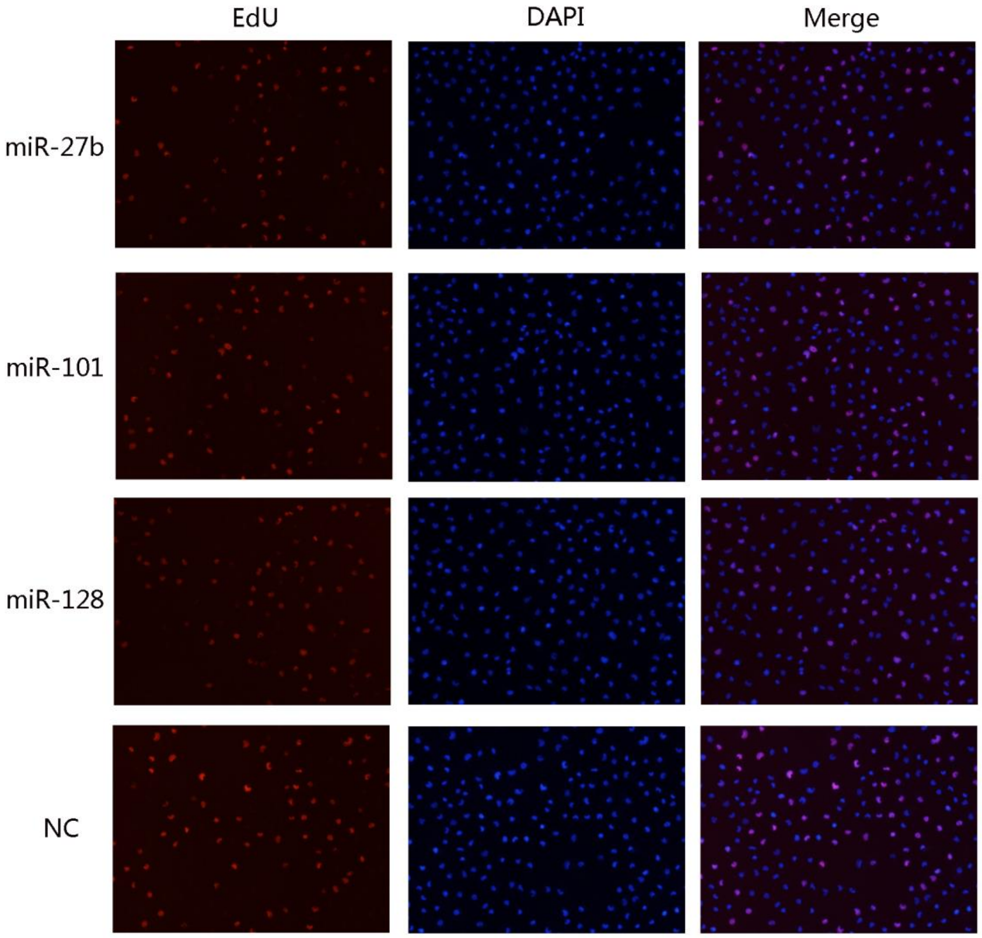

Figure S5. The percentages of proliferating HUVECs were quantified by EdU incorporation experiments in the SGC-7901-treated cells. Decreased proliferation activity of the miRNAs-transfected groups was observed compared to the negative control groups.

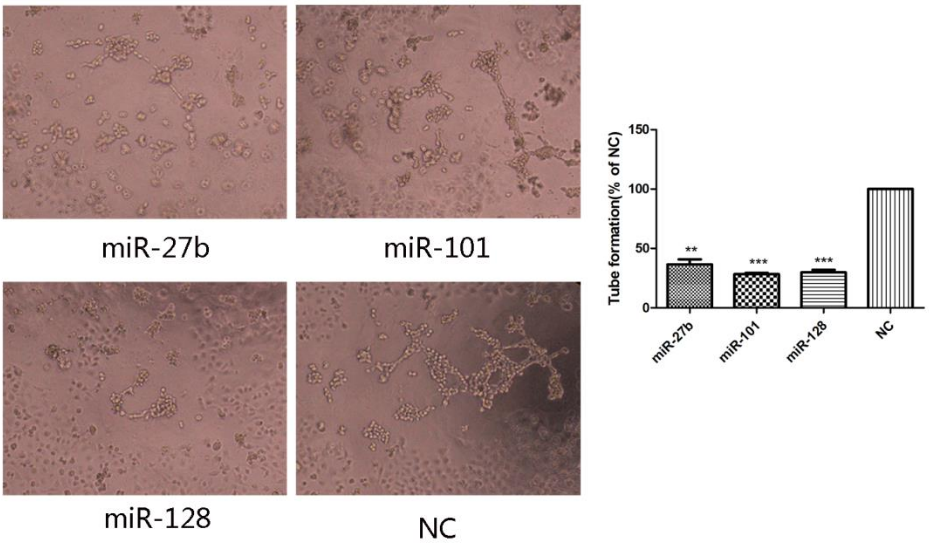

Figure S6. Tube formation of endothelial cells was dramatically inhibited in the transfected groups compared to the negative groups in SGC-7901 cells.

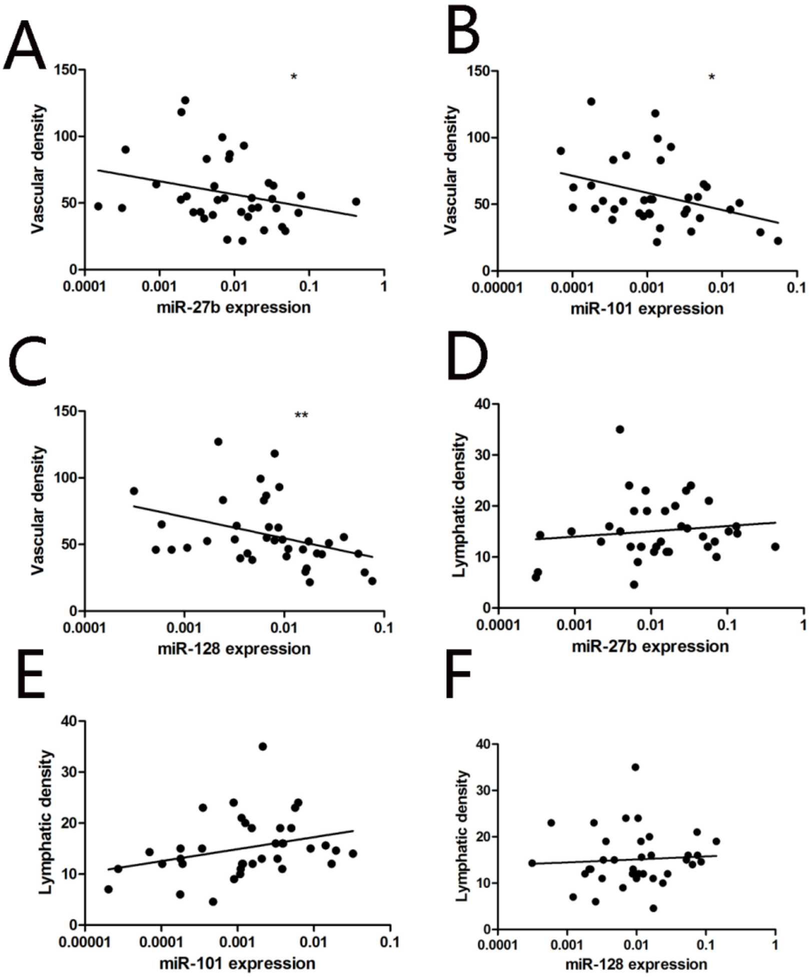

Figure S7. There was a significant inverse correlation between miRNA-27b (A), miR-101(B) or miR-128(C) level and MVD (p = 0.0471, p = 0.0442, or p = 0.0018) respectively, though no correlation between the level of the three miRNAs(D-F) and LVD was found ($p > 0.05$).

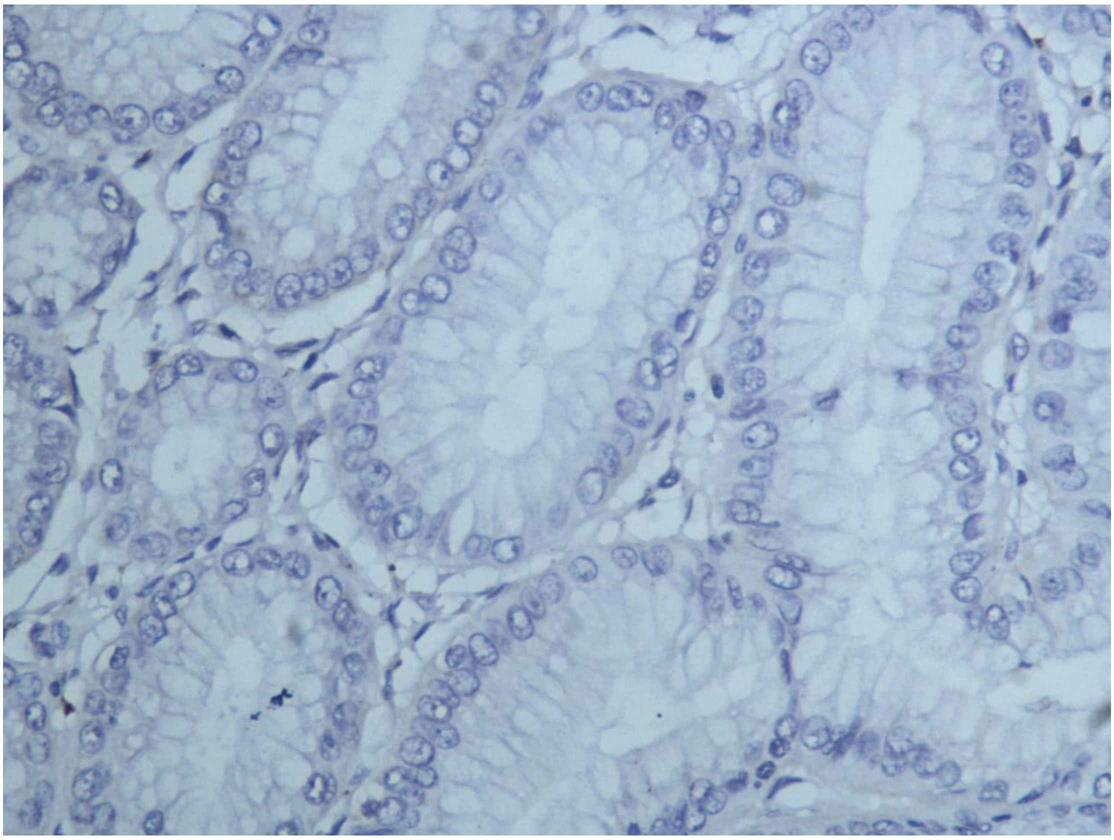

Figure S8. Immunohistochemical staining of VEGF-C in the non-tumorous gastric tissues.
